# The Role of Activating Transcription Factor 3 in Metformin’s Alleviation of Gastrointestinal Injury Induced by Restraint Stress in Mice

**DOI:** 10.3390/ijms241310995

**Published:** 2023-07-01

**Authors:** Bijaya Siwakoti, Te-Sheng Lien, You-Yen Lin, Subhashree Pethaperumal, Shih-Che Hung, Der-Shan Sun, Ching-Feng Cheng, Hsin-Hou Chang

**Affiliations:** 1 Department of Molecular Biology and Human Genetics, Tzu-Chi University, Hualien 97004, Taiwan; bijaya2580@gmail.com (B.S.); alan211@mail.tcu.edu.tw (T.-S.L.); ianlin1985@gmail.com (Y.-Y.L.); subhashreepethaperumal@gmail.com (S.P.); dssun@mail.tcu.edu.tw (D.-S.S.); 2 Institute of Medical Sciences, Tzu-Chi University, Hualien 97004, Taiwan; 102353113@gms.tcu.edu.tw; 3 Department of Pediatrics, Taipei Tzu Chi Hospital, Buddhist Tzu Chi Medical Foundation, Taipei 23142, Taiwan; chengcf@mail.tcu.edu.tw; 4 Institute of Biomedical Sciences, Academia Sinica, Taipei 11529, Taiwan

**Keywords:** metformin, restraint stress, gastrointestinal injury, gastrointestinal leakage, gastrointestinal epithelial cell, apoptosis, activating transcription factor 3, tight junction

## Abstract

Metformin is one of the most commonly used drugs for type 2 diabetes mellitus. In addition to its anti-diabetic property, evidence suggests more potential applications for metformin, such as antiaging, cellular protection, and anti-inflammation. Studies have reported that metformin activates pathways with anti-inflammatory effects, enhances the integrity of gut epithelial tight junctions, and promotes a healthy gut microbiome. These actions contribute to the protective effect of metformin against gastrointestinal (GI) tract injury. However, whether metformin plays a protective role in psychological-stress-associated GI tract injury remains elusive. We aim to elucidate the potential protective effect of metformin on the GI system and develop an effective intervention strategy to counteract GI injury induced by acute psychological stress. By monitoring the levels of GI-nonabsorbable Evans blue dye in the bloodstream, we assessed the progression of GI injury in live mice. Our findings demonstrate that the administration of metformin effectively mitigated GI leakage caused by psychological stress. The GI protective effect of metformin is more potent when used on wild-type mice than on activating-transcription-factor 3 (ATF3)-deficient (*ATF3*^−*/*−^) mice. As such, metformin-mediated rescue was conducted in an ATF3-dependent manner. In addition, metformin-mediated protection is associated with the induction of stress-induced GI mRNA expressions of the stress-induced genes ATF3 and AMP-activated protein kinase. Furthermore, metformin treatment-mediated protection of CD326^+^ GI epithelial cells against stress-induced apoptotic cell death was observed in wild-type but not in *ATF3*^−*/*−^ mice. These results suggest that metformin plays a protective role in stress-induced GI injury and that ATF3 is an essential regulator for metformin-mediated rescue of stress-induced GI tract injury.

## 1. Introduction

Metformin is one of the most widely used drugs and is a first-line therapy in the treatment of type 2 diabetes mellitus. Evidence indicates that metformin inhibits gluconeogenesis, in part mediating through the activation of AMP-activated protein kinase (AMPK), a critical regulator in the modulation of energy metabolism [1,2]. In recent years, in addition to its anti-diabetic property, evidence has suggested more potential applications for metformin, such as antiaging, cellular protection, and anti-inflammation [1,2,3,4]. According to reports, metformin has been shown to activate anti-inflammatory pathways, promote the integrity of gut epithelial tight junctions, and support a healthy gut microbiome, thereby offering protection against gastrointestinal (GI) tract injury [2]. However, the specific role of metformin in protecting against GI tract injury associated with psychological stress remains unclear. Therefore, in this present study, we would like to clarify the putative protective role of metformin in the GI system, thereby developing a feasible intervention strategy to overcome stress-induced GI injury.

Chronic psychological stress has been identified as a risk factor for GI diseases [5,6,7]. Various major psychological and neurodegenerative disorders, including depression, anxiety, autism, bipolar disorder, schizophrenia, and dementia, have been found to be associated with an elevated risk of GI diseases [6,8,9,10,11,12,13]. Consisting of the central nervous system and GI system, the bilateral regulations of the gut–brain axis may explain linkages between GI disorders and psychological stress [14,15]. However, as these associations are generally established in chronic diseases, the linkage between acute psychological stresses with GI injury is less studied, and the mechanism underlying the initiation phase of psychological-stress-induced GI injuries remains elusive.

The restraint-stress model is widely acknowledged as a valuable tool for investigating the physiological, behavioral, and biochemical alterations induced by psychological stress in mice [16,17,18,19]. By assessing the plasma levels of Evans blue dye following oral administration, we can effectively monitor stress-induced GI leakage in real time. Evans blue is a dye that is not typically absorbed by the GI tract and does not normally appear in the circulation of experimental animals [20,21]. In a previous study utilizing the Evans blue dye and the restraint-stress mouse model, we observed that acute restraint stress resulted in GI leakage, which was accompanied by the loss of gut epithelial cells [20]. In this study, we used this mouse model to further investigate the potential therapeutic effect of metformin. Analysis results revealed that treatments with metformin greatly suppressed stress-induced apoptotic cell death in the CD326^+^ epithelial cells of wild-type mice but not in activating-transcription-factor 3 (ATF3)-deficient (*ATF3*^−*/*−^) mice. This suggests that metformin plays a protective role in stress-induced GI injury, and that ATF3 is essential for metformin-mediated rescue of stress-induced GI tract injury.

## 2. Results

### 2.1. Treatments of Metformin-Rescued Restraint-Stress-Induced Platelet Activation and GI Injury

**Figure 1:**
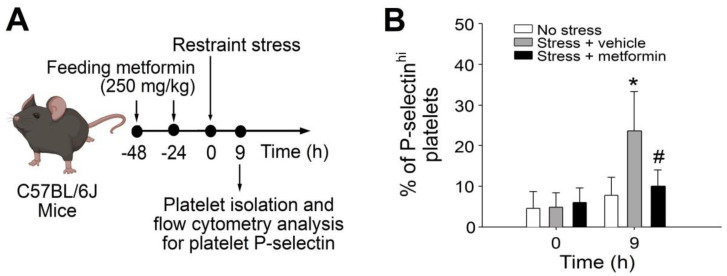
The administration of metformin in mice subjected to restraint stress rescued the up-regulation of circulating P-selectin^hi^ platelets. (**A**) Experimental outline illustrating the restraint-stress mouse model and platelet analysis. (**B**) Flow cytometry analysis was performed to determine the percentage of platelets expressing high surface levels of P-selectin (P-selectin^hi^) in mice subjected to restraint stress, with or without metformin treatment at 0 and 9 h. The error bars represent the standard deviation. * *p* < 0.05 compared with the respective 0-h groups; # *p* < 0.05 compared with the respective stress + vehicle groups. The number of mice used in each group was 6 (three experiments with a total of 6 mice per group).

**Figure 2:**
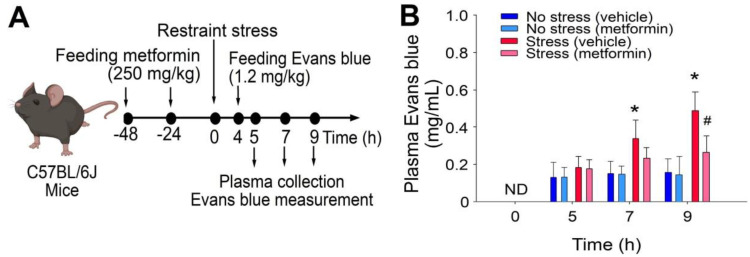
The administration of metformin effectively rescued GI leakage induced by restraint stress in mice. (**A**) Experimental outline depicting the restraint-stress mouse model treated with Evans blue. (**B**) Plasma levels of Evans blue were measured in C57BL/6J mice subjected to restraint stress, with or without metformin treatment at 0, 5, 7, and 9 h. ND: not detected. The error bars represent the standard deviation. * *p* < 0.05 compared with the respective no stress control groups; # *p* < 0.05 compared with the respective vehicle-treated control groups. The number of mice used in each group was 6 (three experiments with a total of 6 mice per group).

Cell death and tissue damage can cause various degrees of platelet activation, and such platelet responses are essential to initiating subsequent anti-inflammatory and repair processes [22,23]. In a previous study, we found that the levels of circulating P-selectin-expressing (P-selectin^hi^) platelets were up-regulated after the restraint stress [21], suggesting that increased levels of P-selectin^hi^ platelets act as a biomarker of restraint-stress-induced tissue injury. In this study, analysis data revealed that metformin treatments ameliorated restraint-stress-induced up-regulation of circulating P-selectin^hi^ platelets (Figure 1A, experiment outline; 1B, % of P-selectin^hi^ platelets). This suggests that metformin may somehow correct the stress-induced abnormalities in the experimental mice. Accordingly, here we would like to further investigate whether metformin treatments can rescue restraint-stress-induced GI leakage. In our study utilizing the restraint-stress mouse model and oral administration of Evans blue dye [20,21], we observed that the administration of metformin effectively alleviated the GI leakage induced by stress (Figure 2).

### 2.2. Treatments of Metformin-Rescued Restraint-Stress-Induced GI Epithelial Cell Apoptosis

**Figure 3:**
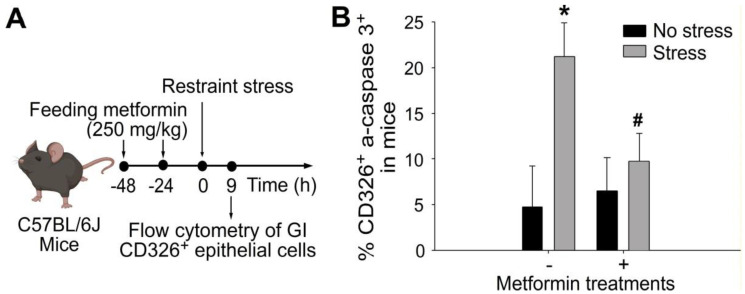
The administration of metformin effectively rescued stress-induced apoptosis of GI CD326^+^ epithelial cells in mice. (**A**) Experimental outline. (**B**) Flow cytometry analysis was performed on GI CD326^+^ epithelial cells from C57BL/6J mice subjected to 9-h restraint stress with or without metformin treatments. CD326 was used as an epithelial cell marker, and active-form caspase 3 (a-caspase 3) was used as an apoptotic cell marker. * *p* < 0.05 compared with the respective no-stress groups; # *p* < 0.05 compared with the respective control groups without metformin treatments (metformin-, vehicle controls). The number of mice used in each group was 6 (three experiments with a total of 6 mice per group).

Acute exposure to restraint stress has been shown to induce apoptotic cell death in GI epithelial cells [20,21]. Flow cytometry analysis demonstrated that treatment with metformin effectively reduced the elevated levels of CD326^+^ and active-form caspase 3^+^ double-positive GI epithelial cells induced by restraint stress (Figure 3). Furthermore, our observations were consistent with these findings, as we observed a significant rescue of GI tight-junction-protein claudin 3 (CLDN3) suppression induced by restraint stress upon metformin treatment through immunohistochemistry staining (Figure S1). These results indicate that metformin exerts a protective effect by inhibiting stress-induced cell death and maintaining the integrity of tight junctions in the gut epithelium during psychological-stress-induced GI injury.

### 2.3. Metformin-Mediated Protection Is Associated with the Induction of GI AMPK and ATF3 mRNA Expression

**Figure 4:**
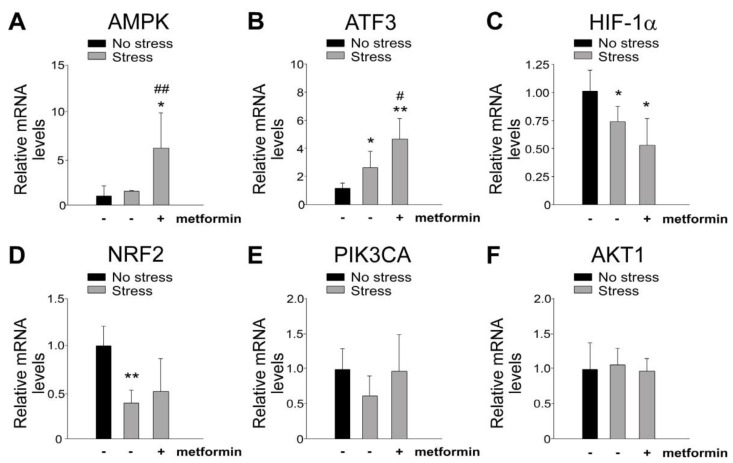
The relative mRNA expression levels of stress-induced genes. Stress-induced genes (A) AMPK, (B) ATF3, (C) HIF-1α, (D) NRF2, (E) PI3KCA, and (F) AKT1 were analyzed using qRT-PCR. The duodenum samples from wild-type C57BL/6J mice were examined with or without 9 h of restraint stress. The mRNA expression levels of the control (no stress) groups were normalized to a fold change of one. Statistical analysis revealed * *p* < 0.05, ** *p* < 0.01 compared with the respective no-stress control groups and # *p* < 0.05, ## *p* < 0.01 compared with the respective stress control groups without metformin treatments (metformin-, vehicle controls). The number of mice used in each group was 4 (two experiments with a total of 4 mice per group).

Stress-induced injuries to the GI tract have been linked to the abnormal regulation of GI mRNA [20,24]. Quantitative reverse transcription polymerase chain reaction (qRT-PCR) analysis was performed to assess the mRNA expression levels of stress-induced genes in mouse GI tissue. The results demonstrated up-regulation of ATF3 and AMPK (Figure 4A,B), down-regulation of hypoxia-inducible factor-1α (HIF-1α) and NFE2-related factor 2 (NRF2) (Figure 4C,D), and no change in phosphoinositide 3-kinase (PI3K) and protein kinase B (AKT) (Figure 4E,F) following restraint stress. Notably, metformin treatment further increased the mRNA expression levels of AMPK and ATF3 (Figure 4A,B), both of which are known to be involved in metformin-mediated cellular protection [1,4,25]. Although the specific involvement of ATF3 in metformin-mediated rescue of stress-induced GI injury remains unclear, these findings suggest a potential role of ATF3 in the protective effects of metformin against such injuries.

### 2.4. ATF3 Deficiency Reduced Metformin-Mediated Rescue in Restraint-Stress-Induced GI Leakage and the Rescue of Stress-Induced GI Epithelial Cell Apoptosis

**Figure 5:**
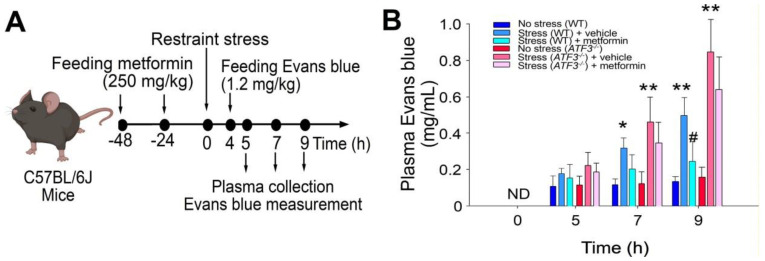
ATF3 deficiency diminishes the efficacy of metformin in rescuing restraint-stress-induced GI injury in mice. (**A**) Experimental design depicting the restraint-stress mouse model with Evans blue treatment. (**B**) Measurement of plasma Evans blue levels in wild-type (WT) and ATF3-gene-knockout (KO; *ATF3*^−*/*−^) mice with or without stress and with or without metformin treatments at different time points (0, 5, 7, 9 h). Statistical analysis revealed * *p* < 0.05, ** *p* < 0.01 compared with the respective no-stress groups, and # *p* < 0.05 compared with the respective control groups without metformin treatments (vehicle controls). The number of mice used in each group was 6 (two experiments with a total of 6 mice per group).

To explore the potential involvement of ATF3 in metformin-mediated rescue of stress-induced GI injury, we utilized *ATF3*^−*/*−^ mice alongside wild-type C57BL/6J mice. Employing a restraint-stress mouse model and administering Evans blue dye orally [20,21], we observed that metformin treatment effectively mitigated stress-induced GI leakage in wild-type mice but not in *ATF3*^−*/*−^ mice (Figure 5A, experiment outline; 5B, plasma Evans blue levels of mice).

**Figure 6:**
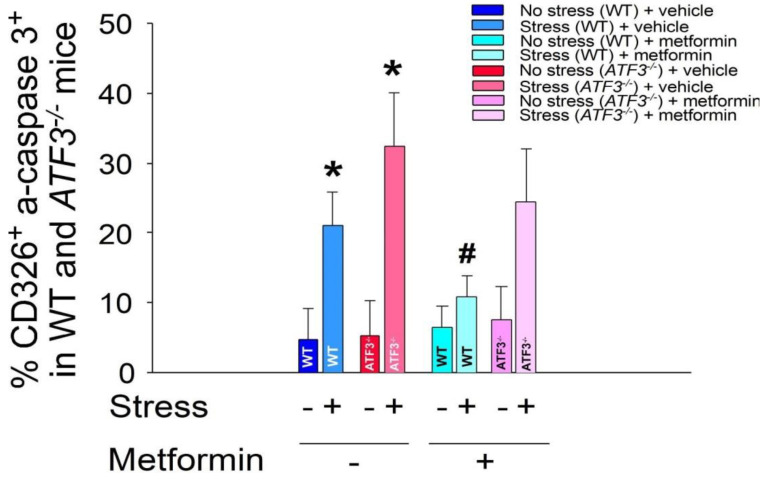
Metformin treatments rescue stress-induced apoptosis of GI CD326^+^ epithelial cells in mice. Flow cytometry analysis was performed on GI CD326^+^ epithelial cells obtained from *ATF3*^−*/*−^ mice, with or without 9-h restraint stress and with or without metformin treatments. CD326 was used as an epithelial cell marker, while active-form caspase 3 (a-caspase 3) served as an apoptotic cell marker. Statistical analysis revealed * *p* < 0.05 compared with the respective no-stress groups, and # *p* < 0.05 compared with the respective control groups without metformin treatments (vehicle controls). The total number of mice used in each group was 6 (three experiments with a total of 6 mice per group).

Furthermore, we employed *ATF3*^−*/*−^ mice to further investigate the role of ATF3 in the metformin-mediated rescue of stress-induced apoptotic cell death in GI epithelial cells. Once again, our findings indicated that metformin treatment suppressed stress-induced apoptotic cell death in the CD326^+^ GI epithelial cells of wild-type mice only, while no such effect was observed in the *ATF3*^−*/*−^ mice (Figure 6). Based on these results, it can be inferred that ATF3 plays a protective role in the metformin-mediated rescue of psychological-stress-induced GI injury.

## 3. Discussion

Metformin, a commonly used drug for type 2 diabetes mellitus, has been recognized for its diverse potential applications beyond its anti-diabetic property [1,2,3,4]. Previous studies have suggested that metformin exhibits anti-aging effects, cellular protection, and anti-inflammatory properties. Notably, metformin has shown protective effects against GI tract injury in various experimental settings [26,27,28,29]. For example, metformin reduced intestinal inflammation by activating the AMPK pathway, contributing to maintaining gut barrier integrity and suppressing inflammation [2,26]. Metformin was shown to improve gut barrier integrity by increasing the expression levels of tight-junction proteins, which are essential for maintaining a strong intestinal barrier [30,31]. Furthermore, metformin has been shown to influence the gut microbiota composition by promoting the growth of beneficial bacteria and inhibiting the growth of harmful microbes, thereby maintaining a healthy gut microbial balance [2,27,29,30]. These findings suggest that metformin’s protective effects on the gut extend beyond glycemic control, making it a potential therapeutic option for gut-related disorders.

Despite the existing evidence of metformin’s protective effects on GI injury, the specific role of metformin in psychological-stress-induced GI injury remains elusive. The impacts of acute psychological stress on the GI system and the potentials for metformin to mitigate the resulting injury have not been thoroughly investigated. Thus, this study aims to address this knowledge gap by clarifying the putative protective role of metformin in the GI system and developing a feasible intervention strategy to overcome acute psychological-stress-induced GI injury.

In this study, GI injury progression was assessed by monitoring the levels of GI-nonabsorbable Evans blue dye in live mice. The findings indicated that metformin treatment improved GI leakage induced by psychological stress, suggesting a potential protective effect against stress-induced GI injury. Notably, the efficacy of metformin in protecting the GI tract was more pronounced in wild-type mice than in mutant mice lacking ATF3, suggesting ATF3’s involvement in metformin-mediated rescue. ATF3 exerts an anti-inflammatory role by suppressing pro-inflammatory cytokines and chemokines, inhibiting NF-κB and AP-1-driven promoters, and promoting immune homeostasis [32,33,34]. It regulates gene expression to mitigate inflammation and maintain immune balance [32,33,34]. ATF3 has also been implicated in the metformin-induced feedback loop between AMPK and GDF15, which contributes to the drug’s anti-diabetic effects [35]. Considering that metformin induces ATF3 expression, ATF3 is considered one of the anti-inflammatory factors induced by metformin in the gut [2]. Therefore, it is reasonable to conclude that ATF3 is involved in the metformin-mediated amelioration of psychological-stress-induced GI leakage in this study.

In addition to its anti-inflammatory properties, ATF3 also plays a critical role in promoting cell survival and protecting against different stresses [32,33,34]. It modulates the expression of genes involved in cell survival pathways [32,33,36]. ATF3 enables cells to adapt to stressful conditions and enhances their resistance to apoptosis, ensuring cell survival and preserving tissue integrity [33,36,37]. Consequently, it is also reasonable to observe that metformin treatment exhibited a protective effect on CD326^+^ GI epithelial cells against stress-induced apoptotic cell death in wild-type mice but not in the ATF3-deficient mice in this study. The GI protective effect of metformin was more prominent in the wild-type mice compared with the ATF3-deficient (*ATF3*^−*/*−^) mice, underscoring the essential role of ATF3 in metformin-mediated rescue.

It poses an intriguing question to determine the specific contributions of ATF3’s anti-inflammatory and pro-survival effects in the rescue of gut leakage induced by restraint stress. Inflammation can induce cellular damage and cell death through the release of reactive oxygen species [38,39], pro-inflammatory cytokines [40], and cytotoxic substances [41]. Conversely, excessive cell death also leads to inflammation [42,43,44,45,46]. Meanwhile, it is known that ATF3 can stimulate autophagy in certain cell types [47,48]. Autophagy may promote a pro-survival and anti-inflammatory cellular response in various conditions [49,50,51]. Given that ATF3 is a stress-responsive gene that is up-regulated to alleviate cellular stress, and its downstream mediators primarily exert anti-inflammatory effects, it becomes challenging to discern between the pro-survival and anti-inflammatory effects of ATF3. Further investigations are warranted to elucidate the distinct pro-survival and anti-inflammatory pathways mediated by ATF3.

It is widely recognized that metformin acts as an activator of AMPK, which has also been demonstrated to possess both anti-inflammatory and pro-survival effects [52,53]. However, the interplay between AMPK and ATF3 in the metformin-mediated rescue of stress-induced GI leakage in mice remains incompletely elucidated. Previous evidence has indicated that ATF3 induction occurs downstream of AMPK activation [54]. Additionally, research has also shown that metformin’s anti-inflammatory effects are achieved through the activation of AMPK and the subsequent induction of ATF3 expression [55]. The inhibitory effects of metformin on the pro-inflammatory cytokine production induced by lipopolysaccharides were nullified when ATF3 was knocked down, accompanied by a reversal of metformin’s suppression of mitogen-activated protein kinase (MAPK) phosphorylation. Conversely, when AMPK was knocked down, all the effects of metformin, including ATF3 induction, inhibition of pro-inflammatory cytokines, and inactivation of MAPK, were blunted [55]. These findings collectively suggest that ATF3 may function as a downstream mediator of AMPK signaling. Consistent with these findings, our present study revealed that metformin-mediated rescue of restraint-stress-induced gut leakage is associated with an increased expression of AMPK and ATF3. Notably, the protective effect of metformin was observed only in wild-type mice and not in *ATF3*^−*/*−^ mice, highlighting the vital role of ATF3 in metformin-mediated rescue.

Although the precise mechanisms underlying metformin-induced AMPK activation and ATF3 stimulation remain unclear, it is postulated that AMPK activation can induce ATF3 expression through various pathways. For example, research has shown that AMPK activation leads to the phosphorylation and activation of transcription factors such as cAMP response element binding protein (CREB) [56], which can bind to the ATF3 gene promoter and enhance its transcription [57]. Additionally, AMPK has interactions with the mammalian target of rapamycin (mTOR) pathway [58], which is involved in the regulation of ATF3 [59] and MAPKs [60]. Notably, MAPKs are known regulators of ATF3 [61]. These findings suggest that AMPK activation triggers ATF3 activation through a combination of transcriptional regulation, modulation of signaling pathways, and post-translational modifications. However, these regulatory mechanisms have not yet been fully established in GI cells, emphasizing the need for further investigations to unravel the precise regulation and underlying mechanisms of metformin-mediated stimulation of AMPK and ATF3.

Despite the conducted experiments, it is important to acknowledge several limitations in the present study. Firstly, the investigation was confined to a mouse model, cautioning against direct extrapolation of the findings to humans. The intricate pathophysiology of stress-induced GI injury may vary across species, necessitating further research involving human subjects. Additionally, the study primarily focused on ATF3 as a mediator of metformin’s protective effects, leaving room for additional exploration of the specific molecular mechanisms at play. Unraveling the downstream signaling pathways and interactions underlying metformin’s actions on GI injury is crucial for a comprehensive understanding of its therapeutic potential. Moreover, it is imperative to explore alternative targets and pathways for intervention. While the study underscores the significance of ATF3, it is plausible that other transcription factors and regulatory molecules also contribute to metformin’s protective effects. Investigating these factors may reveal novel therapeutic targets and facilitate the development of more effective interventions. Ultimately, considering the translational implications, the study suggests that metformin shows promise as an intervention for stress-induced GI injury. The demonstrated protective effects and the involvement of ATF3 lay the groundwork for further preclinical and clinical investigations. Translating these findings into clinical practice could lead to the formulation of targeted therapeutic strategies that alleviate the GI injury associated with acute psychological stress, benefiting individuals at risk of stress-related GI disorders.

In conclusion, this study sheds light on the protective role of metformin in stress-induced GI injury and emphasizes the importance of ATF3 as a modulator of its effects. The findings underscore the potential clinical applications of metformin as an intervention for stress-related GI disorders. However, further research is warranted to elucidate the underlying molecular mechanisms, validate the translational potential in humans, and explore additional stress models. Ultimately, the knowledge gained from these investigations may pave the way for the development of targeted therapeutic strategies to alleviate GI injury associated with acute psychological stress, benefiting individuals at risk of stress-related GI disorders.

## 4. Materials and Methods

### 4.1. Laboratory Mice

Male C57BL/6J mice, wild-type and aged between 8 and 12 weeks, were acquired from the National Laboratory Animal Center in Taipei, Taiwan [62,63,64,65,66,67,68]. The genetically deficient ATF3 KO (*ATF3^−/−^*) mice with a C57BL/6J background were generously provided by Dr. Tsonwin Hai [20,69,70]. To generate ATF3 KO mice, an ATF3 genomic DNA clone was isolated from a 129SVJ library, and the ATF3 KO mutants were generated in the 129SVJ background [69]. To confirm the presence of ATF3 KO in the mice, the KO allele was distinguished from the wild-type allele using PCR. In the PCR analysis, three primers were utilized: 5′-AGAGCTTCAGCAATGGTTTGC-3′, 5′-TGAAGAAGGTAAACACACCGTG-3′, and 5′-ATCAGCAGCCTCTGTTCCAC-3′ [69]. From this analysis, a 329 bp PCR product was obtained from the wild-type mice, a 236 bp PCR product was obtained from the ATF3 KO (*ATF3^−/−^*) mice, and both a 236 bp and a 329 bp PCR product were obtained from the *ATF3^+/−^* heterozygotes [69]. Additionally, we regularly checked ATF3 RNA and protein expression through qRT-PCR and/or flow cytometry [20]. To establish congenic KO mice in the C57BL/6 background, *ATF3*^−*/*−^ mice were backcrossed with wild-type C57Bl/6J mice for more than 10 generations. The animals, including approximately 240 wild-type mice and 100 *ATF3*^−*/*−^ mice, were kept in a specific pathogen-free facility at the Animal Center of Tzu-Chi University. The facility maintained controlled lighting and temperature conditions, and the animals were provided with free access to food and filtered water. All procedures involving the experimental animals were conducted in accordance with the approved guidelines and protocols of the Animal Care and Use Committee of Tzu-Chi University, Hualien, Taiwan (approval ID: 110024).

### 4.2. Induction, Reversal and Measurement of Stress-Induced GI Leakage

A mouse model of restraint-stress-induced GI leakage, utilizing the administration of Evans blue dye, was established following the previously described protocols [20]. Male mice within the age range of 12–16 weeks and with a body weight exceeding 25g were selected for the experiment. To induce restraint stress, the mice were confined in a 50-mL plastic Falcon tube for a duration of 9 h, as described in previous studies [20,71,72]. To ensure adequate air supply, small holes were made at the tapering end of the Falcon tube. Blood samples (50 µL) were collected at various time points (0, 5, 7, and 9 h) throughout the experiment following the stress challenge. The metformin tablets (N,N-dimethylbiguanide, 500mg each, Shou Chan Industrial Co., Nantou, Taiwan) were crushed into powder and mixed with pure water (Milli-Q, Merck Millipore, Burlington, MA, USA). This metformin solution was administered orally (250 mg/kg/day for 2 days) to the mice using a steel feeding tube before subjecting them to restraint stress. The administration of metformin occurred 48 and 24 h prior to the initiation of the restraint-stress challenges in the mice. The mice were orally administered Evans blue (1.2 g/kg, Santa Cruz Biotechnology, Santa Cruz, CA, USA) using a feeding tube four hours after the initiation of the stress challenge [20]. Blood plasma was obtained by collecting blood in an Eppendorf tube and combining it with an equal volume of an anticoagulant citrate dextrose solution to prevent clotting [43,44,45]. The collected plasma was transferred to 96-well plates, and the concentration of Evans blue was determined using a full-spectrum analyzer (Multiskan Spectrum, Thermo Fisher Scientific, Waltham, MA, USA) at a wavelength of 620 nm.

### 4.3. Quantitative Reverse Transcription Polymerase Chain Reaction (qRT-PCR)

#### 4.3.1. RNA and cDNA Preparation

RNA isolation and cDNA preparation were conducted following established protocols [20]. Mouse duodenum samples (1 cm, beginning immediately after the pyloric portion of the stomach) were isolated, washed with phosphate-buffered saline (PBS), and then dissolved in Trizol (Ambion, Thermo Fisher Scientific) after 9 h of stress challenge. Following standard isolation procedures, the concentration of RNA was determined using a NanoDrop spectrophotometer (Thermo Fisher Scientific). Subsequently, 1 μg of the isolated RNA was used to synthesize complementary DNA (cDNA) using the iScript cDNA Synthesis Kit (Bio-Rad Laboratories, Hercules, CA, USA). The resulting cDNA was utilized for PCR and qRT-PCR analyses, and the samples were stored at −20 °C until further use [20].

#### 4.3.2. qRT-PCR Analyses

To analyze the expression of stress-induced genes involved in mucosal homeostasis and intestinal integrity, such as ATF3, and AMPK [20,73,74,75,76], qRT-PCR was performed on GI tissues. For each reaction, 2 µL of cDNA was mixed with 10 µL of SYBR Green (Thermo Fisher Scientific), 0.5 µL each of forward and reverse primers, and 7 µL of pure water (Milli-Q, Merck Millipore). The cDNA was then quantified using a real-time reverse transcription linkage instrument (StepOnePlus Real-Time PCR System, Thermo Fisher Scientific), with varying annealing temperatures according to the primer specifications. Triplicate runs were performed for each sample, and the average cycle threshold (Ct) values were used to calculate the relative expression using the 2^−ΔΔCT^ method, with GAPDH (glyceraldehyde-3-phosphate dehydrogenase) as the internal control. The primer sequences can be found in Supplementary Table S1.

### 4.4. Flow Cytometry Analysis

Flow cytometry analysis of platelet surface P-selectin and CD41 (integrin αIIb) expression levels before and after stress was performed using FACScalibur (BD Biosciences, Franklin Lakes, NJ, USA) and Gallios (Beckman Coulter Life Sciences) flow cytometers [20,77,78]. For staining, anti-mouse phycoerythrin-conjugated P-selectin Ig (eBioscience, Thermo Fisher Scientific) [45] and Alexa Fluor 488-conjugated anti-mouse CD41 Ig (clone MWReg30 [79]; Biolegend, San Diego, CA, USA) were used to stain platelet-surface P-selectin and CD41, respectively. Mouse GI epithelial cells were analyzed following previously described methods [20]. Duodenum samples from mice (6 cm; starting immediately after the pyloric portion of the stomach; washed with PBS) were cut into small pieces and incubated with serum-free cell culture medium containing collagenase D (Sigma-Aldrich, Burlington, MA, USA; 1 mg/mL) for 30 min in a 15 mL Falcon tube at 37°C with shaking (OSI 500, Kansin Instruments, New Taipei City, Taiwan) after 9 h of restraint stress. The dissociation of mouse GI epithelial cells from cell clusters and tissue pellets was achieved by incubating the samples with 2 mL of non-enzymatic cell-dissociation solution (Sigma-Aldrich) for 10 min at 25°C. After washing, the dissociated cells were fixed with 500 µL of a fixation buffer (Cytofix, BD Biosciences, San Jose, CA, USA) and incubated at 25°C for 20 min. The samples were then centrifuged at 300 g for 5 min. Following washing (Perm/Wash buffer, BD Biosciences) and blocking (5% bovine serum albumin in RPMI), the cells were stained with CD326 (a gut epithelial cell marker [80,81]) and cell-death markers (anti-CD326 epithelial cell marker antibody, BioLegend; anti-cleaved caspase-3 antibody, Cell Signaling Technology, Danvers, MA, USA) [20]. Flow cytometry analysis (Gallios, Beckman Coulter Life Sciences, Brea, CA, USA) was performed on the samples to quantify stress-induced GI epithelial cell apoptosis, as previously described [20].

### 4.5. Confocal Microscopy for Immunohistochemistry Samples

Following 9 h of stress, duodenum samples measuring 6 cm in length, starting immediately after the pyloric portion of the stomach and washed with PBS, were collected. The samples underwent a series of steps as described below. First, the samples underwent three rinses with 1 mL of ice-cold PBS and were then treated twice with Bouin’s reagent (1 mL), which consisted of 50% Milli-Q pure water, 45% absolute ethanol, and 5% acetic acid. A longitudinal incision was made in the duodenum using a surgical blade, followed by two additional washes with PBS. The duodenal tissue was then subsequently dehydrated overnight in organic solvents. After dehydration, the tissue was embedded in hot wax within a metal cassette, which was then allowed to cool and solidify the wax. The metal cassette was removed, and standard protocols for paraffin embedding, removal, antigen retrieval, washes (using TBS buffer), and protein blocking (with 5% BSA) were followed [64,82]. The sectioned tissues were subjected to fluorescent labeling using specific antibodies, including an anti-claudin-3 antibody (Thermo Fisher Scientific), and 4’,6-diamidino-2-phenylindole (DAPI, Sigma-Aldrich) for staining. Following staining, cover slips were mounted using a 30 µL Mowiol 4-88 mounting solution, composed of 2.5% 1,4-diazabicyclo-octane, 10% Mowiol 4-88, 25% glycerol, and 0.1 M Tris-HCl. Confocal microscopy (LSM 800, ZEN 2.1 software, Carl Zeiss, Jena, Germany) was utilized for fluorescence imaging. The Zeiss confocal microscope was equipped with a Plan-Apochromat 10×/0.45, Plan-Apochromat 20×/0.8, and Plan-Apochromat 40×/1.3 Oil DIC (UV) VIS-IR lens. DAPI-labeled nuclei were excited using a 405-nm laser, and the signals were collected with an SP 470 filter. The green fluorescence-labeled structures were excited using a 488-nm laser, and the fluorescence signal was collected with an SP 545 filter. At least three independent images were analyzed in each section, and the quantified results of fluorescence intensity were obtained using ImageJ 1.52a software [43,64].

### 4.6. Statistical Analysis

The data obtained from the experiments were analyzed using Microsoft Office Excel 2003. The results are presented as mean ± standard deviation. Statistical significance was determined using one-way analysis of variance (ANOVA) followed by a post-hoc Bonferroni-corrected *t*-test. A significance level of α = 0.05 was used as the threshold for determining statistical significance, corresponding to a probability of type 1 error.

## Data Availability

The datasets utilized and analyzed in this study can be obtained and are available from the corresponding author upon a reasonable request.

## Supplementary Materials

The following supporting information can be downloaded at: https://www.mdpi.com/article/10.3390/ijms241310995/s1.
