# Supplementary material for: The Role of Activating Transcription Factor 3 in Metformin’s Alleviation of Gastrointestinal Injury Induced by Restraint Stress in Mice
Source: Int J Mol Sci. 2023 Jul 1;24(13):10995. doi: 10.3390/ijms241310995 (PMC10342009; doi:10.3390/ijms241310995)
Supplement: Supplementary file 1 [file ijms-24-10995-s001.zip › ijms-2448775-supplementary.pdf]

Supplementary information

The Role of Activating Transcription Factor 3 in Metformin's Alleviation of Gastrointestinal Injury Induced by Restraint Stress in Mice

Bijaya Siwakoti [1], Te-Sheng Lien [1], You-Yen Lin[1], Subhashree Pethaperumal[1], Shih-Che Hung [2], Der-Shan Sun [1,2], Ching-Feng Cheng [3,4], and Hsin-Hou Chang [1,2]*

[1] Department of Molecular Biology and Human Genetics, Tzu-Chi University, Hualien 97004, Taiwan;
bijaya2580@gmail.com (B.S.); alan211@mail.tcu.edu.tw (T.-S.L.);
ianlin1985@gmail.com (Y.-Y.L.);
subhashreepethaperumal@gmail.com (S.P.); dssun@mail.tcu.edu.tw (D.-S.S.)
[2] Institute of Medical Sciences, Tzu-Chi University, Hualien 97004, Taiwan;
102353113@gms.tcu.edu.tw
[3] Department of Pediatrics, Taipei Tzu Chi Hospital, Buddhist Tzu Chi Medical Foundation, Taipei 23142, Taiwan; chengcf@mail.tcu.edu.tw
[4] Institute of Biomedical Sciences, Academia Sinica, Taipei 11529, Taiwan
* Correspondence: hhchang@mail.tcu.edu.tw; Tel.: +886-3-8565301 (ext. 2667)

Running title: Metformin protects against gastrointestinal injury through ATF3

Key words: Metformin, restraint stress, gastrointestinal injury, gastrointestinal leakage, gastrointestinal epithelial cell, apoptosis, activating transcription factor 3, tight junction

* To whom correspondence and reprint requests should be addressed.

Hsin-Hou Chang Ph.D. Room D407, Tzu-Chi University, No. 701, Section 3, Chung-Yang Road, Hualien 97004, Taiwan. Tel: 886-3-8565301 ext 2667. Fax: 886-3-8578386.
E-mail: hhchang@mail.tcu.edu.tw

Figures S1: page 2

Table S1: page 3

Figure S1

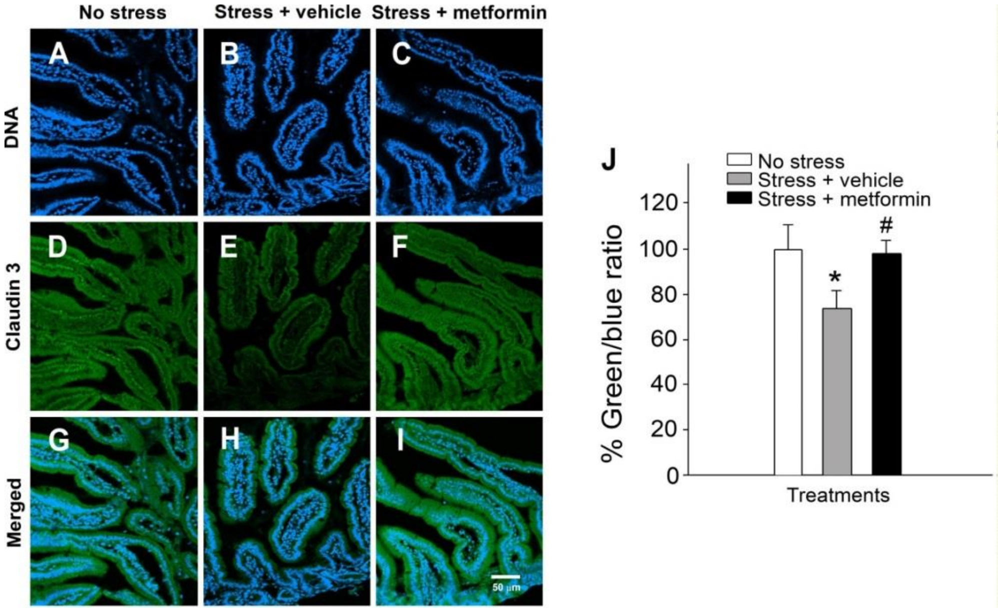

Figure S1. Immunohistochemistry (IHC) staining of GI tight junction protein claudin 3 (CLDN3) visualized through confocal microscopy. (A-I) Representative IHC images of mouse duodenal tissues are displayed. (A-C) DAPI staining shows DNA signals. (D-F) Signals derived from anti-CLDN3 antibody staining. (G-I) Merged signals display DNA and CLDN3. (A, D, G) Images derived from the no stress groups; (B, E, H) images from stress groups treated with vehicle; (C, F, I) Images from stress groups treated with metformin. (J) Quantitative results derived from three images per condition. * $P < 0.05$, compared to the no stress groups; # $P < 0.05$, compared to the vehicle-treated groups. N = 3. To quantify CLDN3 expression, in the merged images, the mean fluorescence intensity of CLDN3 (green) was normalized by dividing it with the mean fluorescence intensity of DNA (blue), which is proportional to the cell number. The average green/blue signal ratio of the no stress groups was normalized to 100 %. Scale bar: 50 µm.

Table S1

List of primers used in the qRT-PCR analysis of this report

**Gene symbol**	**Forward primer (5'→3')**	**Reverse primer (5'→3')**	**Product size (bp)**	**Temperatur e (°C)**
**GAPDH**	TCAACAGCAACTC CCACTCTTCCA	ACCCTGTTGCTG TAGCCGTATTCA	115	55
**PRKAA1 (AMPK)**	GAAACCTGAGAA CGTCCTGC	GCCTGCGTACAA TCTTCCTG	157	54
**ATF3**	AGTGACAGCATG AGCCCTCT	GCAGCACTGACC TGATCAAA	179	60
**HIF1-α**	CCAGCAGACCCA GTTACAGA	TGAGTGCCACTG TATGCTGA	186	55
**NRF 2**	GAGCTAGATAGT GCCCCTGG	CAGGACTCACGG GAACTTCT	169	52
**PIK3CA**	CTTGACCTTCGCA TGCTACC	AGCCATTGATGC AGTGTGTG	158	54
**AKT 1**	AAGGACGGTGCC ACTATGAA	TCCTGGTTGTAG AAGGGCAG	158	55
